# Decreased in vivo glutamate/GABA ratio correlates with the social behavior deficit in a mouse model of autism spectrum disorder

**DOI:** 10.1186/s13041-022-00904-z

**Published:** 2022-02-19

**Authors:** Gaeun Park, Se Jin Jeon, In Ok Ko, Ji Hwan Park, Kyo Chul Lee, Min-Sik Kim, Chan Young Shin, Hyeonjin Kim, Yong-Seok Lee

**Affiliations:** 1 Department of Biomedical Sciences, BK21 Four Biomedical Science Program, Seoul National University College of Medicine, Seoul, Republic of Korea; 2 Department of Physiology, Seoul National University College of Medicine, Seoul, Republic of Korea; 3 School of Medicine and Center for Neuroscience Research, Konkuk University, Seoul, Republic of Korea; 4 Division of Applied RI, Korea Institute Radiological and Medical Sciences, Seoul, Republic of Korea; 5 Department of New Biology, Daegu Gyeongbuk Institute of Science and Technology (DGIST), Daegu, Republic of Korea; 6 Department of Medical Sciences, Seoul National University College of Medicine, Seoul, Republic of Korea; 7 Neuroscience Research Institute, Seoul National University College of Medicine, Seoul, Republic of Korea; 8 Wide River Institute of Immunology, Seoul National University, Hongcheon, Republic of Korea

**Keywords:** Autism spectrum disorder, E/I balance, Glu/GABA, Creatine, Sociability, Magnetic resonance spectroscopy

## Abstract

To diagnose autism spectrum disorder (ASD), researchers have sought biomarkers whose alterations correlate with the susceptibility to ASD. However, biomarkers closely related to the pathophysiology of ASD are lacking. Even though excitation/inhibition (E/I) imbalance has been suggested as an underlying mechanism of ASD, few studies have investigated the actual ratio of glutamate (Glu) to γ-aminobutyric acid (GABA) concentration in vivo. Moreover, there are controversies in the directions of E/I ratio alterations even in extensively studied ASD animal models. Here, using proton magnetic resonance spectroscopy (^1^H-MRS) at 9.4T, we found significant differences in the levels of different metabolites or their ratios in the prefrontal cortex and hippocampus of *Cntnap2*^−/−^ mice compared to their wild-type littermates. The Glu/GABA ratio, N-acetylaspartate (NAA)/total creatine (tCr) ratio, and tCr level in the prefrontal cortex were significantly different in *Cntnap2*^−/−^ mice compared to those in wild-type mice, and they significantly correlated with the sociability of mice. Moreover, receiver operating characteristic (ROC) analyses indicated high specificity and selectivity of these metabolites in discriminating genotypes. These results suggest that the lowered Glu/GABA ratio in the prefrontal cortex along with the changes in the other metabolites might contribute to the social behavior deficit in *Cntnap2*^−/−^ mice. Our results also demonstrate the utility of ^1^H-MRS in investigating the underlying mechanisms or the diagnosis of ASD.

**Supplementary Information:**

The online version contains supplementary material available at 10.1186/s13041-022-00904-z.

## Introduction

Autism spectrum disorder (ASD) is a neurodevelopmental disorder that exhibits a range of behavioral symptoms, such as restricted interests and repetitive behaviors, and impaired social interactions [1]. For a swift medical intervention for ASD patients, diverse approaches to diagnose ASD have been undertaken that have lowered the minimum age for ASD diagnosis to 18 months from birth [2]. However, finding prominent biomarkers for ASD remains the focus of clinical professionals and researchers, as early intervention can successfully alleviate phenotypical severity before the pervasive symptoms of ASD may arise [3, 4].

Classically, ASD can be diagnosed with the patients’ behavioral abnormalities as they are unable to react to socially relevant stimuli [5]. Metabolic biomarkers, such as blood serotonin levels, have been examined, as many metabolic disorders are possibly associated with autistic phenotypes [6, 7]. Non-invasive, but more direct biomarkers of ASD using neuroimaging have recently been identified. Functional connectivity between different regions has been observed and used to diagnose ASD [8]. The theta–gamma frequency in the frontal lobe is high in children with ASD, but not in typically developing (TD) groups, as shown by electroencephalogram (EEG) analytics [9]. Magnetic resonance imaging (MRI) or positron emission tomography (PET) facilitates in discerning subtle changes in region-specific brain volume, often shown in neurodevelopmental disorders [10]. Proton magnetic resonance spectroscopy (^1^H-MRS) can directly observe the abundance of metabolites in specific brain regions and has been suggested as an imaging tool to diagnose ASD [11].

The excitation/inhibition (E/I) imbalance observed in different brain regions such as the prefrontal cortex (PFC) has been shown to be critical for social deficits in various ASD mouse models [1, 12, 13]. Along with their genetic heterogeneities, different ASD mouse models exhibit diverse alterations in the excitatory and inhibitory synaptic functions in the neocortex and/or hippocampus (HPC) [14, 15]. For example, the E/I ratio decreased with reduced mEPSC amplitude in cortical layer 5 of MECP2 mutant mice or increased mIPSC frequency in layer 2/3 of Neuroligin3^R451C^ mutant mice [16, 17]. However, other studies also exhibit increased E/I ratio as reduced spontaneous GABAergic inhibitory transmission in BTBR mice or increased mEPSC in the PFC of VPA-exposed mice [18, 19]. In particular, although alterations in E/I balance have also been reported in *contactin-associated protein-like 2* knockout (*Cntnap2*^−/−^) mice [13, 20–23], the directions of E/I ratio alterations are inconsistent depending on the literatures [13, 20, 21]. Since the previous studies were performed using brain slices, we hypothesized that the discrepancy might be due to the ex vivo experimental conditions, such as brain regions and recording conditions. Therefore, we examined the E/I ratio and other metabolites in multiple brain regions of *Cntnap2*^−/−^ mice using the MRS technique in the present study.

## Methods

### Mice

*Cntnap2* knockout (*Cntnap2*^−/−^) mice were a generous gift from Dr. Daniel H. Geschwind (University of California Los Angeles). Twenty-week-old male *Cntnap2*^+/+^ and *Cntnap2*^−/−^ mice were used for the behavioral tests and 9.4T ^1^H-MRS. The same mice were used for behavioral tests and MRS. Mice were housed with a fixed 12-h light/dark cycle, and food and water were provided ad libitum.

### Social behavior test

Three-chamber social behavior tests were performed as described previously [19, 24]. The tests comprised three sessions: (1) habituation, (2) social preference test, and (3) social recognition test. During the habituation session, each subject mouse was introduced to the 3-chamber behavior apparatus and allowed to move freely for 5 min. After habituation, a novel target conspecific was introduced to a wired cup on one side of the chamber, while the other side of the chamber remained empty. Each location for a novel conspecific and empty cup was counterbalanced. After 10 min of the social preference test, another novel conspecific was introduced to the empty cup for the social recognition test. The subject mouse was allowed to explore conspecifics for another 10 min during the social recognition test. Behavior sessions were recorded and analyzed manually, while the experimenters were blinded to the genotype of the subject mice. To normalize the variation among subject mice, all the behavior data were represented by the percentage of time spent investigating one side out of the summation of investigation times for both sides. The preference index (PI) was calculated using the following equations: ET_M_, exploration time for a mouse; ET_E_, exploration time for an empty cup; ET_N_, exploration time for a novel mouse; ET_F_, exploration time for a familiar mouse).

$$PI for social preference = \frac{{ET}_{M}-{ET}_{E} }{{ET}_{M}+{ET}_{E}}$$

$$PI for social recognition = \frac{{ET}_{N}-{ET}_{F} }{{ET}_{N}+{ET}_{F}}$$

### Magnetic resonance spectroscopy (MRS)

**Fig. 1:**
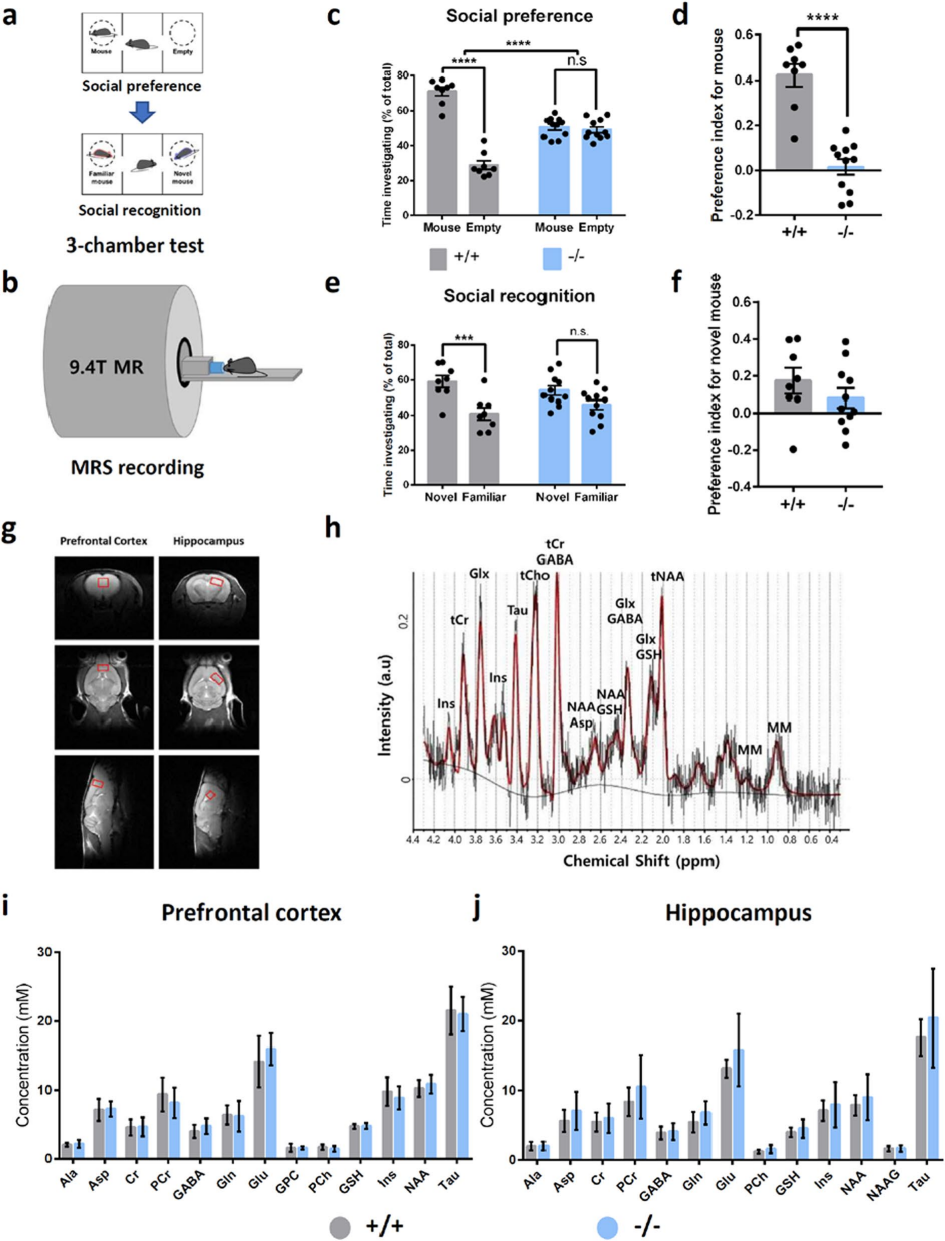
*Cntnap*2-null mice show deficits in social behaviors. **a** Schematic diagram for three-chamber social behavior tests. Social preference test was followed by social recognition test. **b** Schematic diagram for 9.4T ^1^H-magnetic resonance spectroscopy (MRS) for mouse. **c** In the social preference test, *Cntnap2*^−/−^ mice did not showed a significant preference towards target conspecifics versus an empty chamber, while *Cntnap2*^+/+^ mice showed a significant preference towards target conspecifics. Two-way ANOVA: *Cntnap2*^+/+^, n = 9 mice, *Cntnap2*^−/−^, n = 11 mice, interaction between genotype × target, *F*_1, 34_ = 97.71, ****P < 0.0001; Sidak’s multiple comparisons test: *Cntnap2*^+/+^, mouse versus object *t*_34_ = 13.49, ****P < 0.0001; *Cntnap2*^−/−^, mouse versus object *t*_34_ = 0.5863, *P* = 0.5863. n.s., not significant. **d** Deletion of *Cntnap2* gene significantly affect the social preference index in *Cntnap2*^−/−^ mice. Two-tailed unpaired t-test with Welch’s correction, ****P < 0.0001. **e** In the social recognition test, *Cntnap2*^−/−^ mice did not showed a significant preference towards novel conspecifics versus familiar conspecifics, while *Cntnap2*^+/+^ mice showed a significant preference towards novel conspecifics. Two-way ANOVA: *Cntnap2*^+/+^, n = 9 mice, *Cntnap2*^−/−^, n = 11 mice, interaction between genotype × social target, *F*_1, 34_ = 2.882, *P* = 0.0987; Sidak’s multiple comparisons test: *Cntnap2*^+/+^, novel mouse versus familiar mouse *t*_34_ = 3.954, ***P = 0.0007; *Cntnap2*^−/−^, novel mouse versus familiar mouse *t*_34_ = 2.02, *P* = 0.0999. n.s., not significant. **f** Deletion of *Cntnap2* gene did not affect the social preference index for novel mouse in *Cntnap2*^−/−^ mice. Two-tailed unpaired t-test with Welch’s correction, *P* = 0.3010. **g** Example voxel images targeting prefrontal cortex and hippocampus of mouse as coronal, axial and sagittal planes. **h** Example image of 9.4T ^1^H-MR spectra shown as a black trace. Red trace shows the result of LC model fit. **(i)** Comparison of metabolite concentrations monitored by 9.4T ^1^H-MRS in the prefrontal cortex. Metabolites which number of individuals with CRLB ≤ 50% is greater than 5 were included for statistical analysis (Ala: *Cntnap2*^+/+^, n = 8; *Cntnap2*^−/−^, n = 10; Asp: *Cntnap2*^+/+^, n = 9; *Cntnap2*^−/−^, n = 11; Cr: *Cntnap2*^+/+^, n = 5; *Cntnap2*^−/−^, n = 10; PCr: *Cntnap2*^+/+^, n = 9; *Cntnap2*^−/−^, n = 11; GABA: *Cntnap2*^+/+^, n = 8; *Cntnap2*^−/−^, n = 11; Gln: *Cntnap2*^+/+^, n = 9; *Cntnap2*^−/−^, n = 11; Glu: *Cntnap2*^+/+^, n = 9; *Cntnap2*^−/−^, n = 11; GPC: *Cntnap2*^+/+^, n = 8; *Cntnap2*^−/−^, n = 9; PCh: *Cntnap2*^+/+^, n = 7; *Cntnap2*^−/−^, n = 10; GSH: *Cntnap2*^+/+^, n = 9; *Cntnap2*^−/−^, n = 11; Ins: *Cntnap2*^+/+^, n = 9; *Cntnap2*^−/−^, n = 11; NAA: *Cntnap2*^+/+^, n = 9; *Cntnap2*^−/−^, n = 11; Tau: *Cntnap2*^+/+^, n = 9; *Cntnap2*^−/−^, n = 11). **j** Comparison of metabolite concentrations monitored by 9.4T ^1^H-MRS in the hippocampus. Metabolites which number of individuals with CRLB ≤ 50% is greater than 5 were included for statistical analysis (Ala: *Cntnap2*^+/+^, n = 9; *Cntnap2*^−/−^, n = 10; Asp: *Cntnap2*^+/+^, n = 9; *Cntnap2*^−/−^, n = 11; Cr: *Cntnap2*^+/+^, n = 9; *Cntnap2*^−/−^, n = 10; PCr: *Cntnap2*^+/+^, n = 9; *Cntnap2*^−/−^, n = 11; GABA: *Cntnap2*^+/+^, n = 9; *Cntnap2*^−/−^, n = 11; Gln: *Cntnap2*^+/+^, n = 9; *Cntnap2*^−/−^, n = 11; Glu: *Cntnap2*^+/+^, n = 9; *Cntnap2*^−/−^, n = 11; PCh: *Cntnap2*^+/+^, n = 8; *Cntnap2*^−/−^, n = 9; GSH: *Cntnap2*^+/+^, n = 9; *Cntnap2*^−/−^, n = 11; Ins: *Cntnap2*^+/+^, n = 9; *Cntnap2*^−/−^, n = 11; NAA: *Cntnap2*^+/+^, n = 9; *Cntnap2*^−/−^, n = 11; NAAG: *Cntnap2*^+/+^, n = 8; *Cntnap2*^−/−^, n = 9; Tau: *Cntnap2*^+/+^, n = 9; *Cntnap2*^−/−^, n = 11)

MRS data were acquired on a 9.4T animal MR system (Agilent Technologies, USA) using a volume coil (inner diameter = 72 mm) for RF transmission and a surface coil (phased array 2-channel for mouse brain) for signal reception (Rapid Biomedical GmbH, Rimpar, Germany). T2-weighted scout images were acquired in the axial, coronal, and sagittal planes using a multi-slice turbo spin-echo sequence (repetition time [TR] = 3500 ms, effective echo time [TE_eff_] = 30 ms, echo train length [ETL] = 6, field of view [FOV] = 20 × 20 mm^2^, matrix size = 128 × 128, slice thickness = 0.8 mm, number of averages (NA) = 1, and scan time = 1 min 24 s). The spectroscopic voxel was positioned in the prefrontal cortex (1.6 × 1.2 × 1.5 mm^3^) and hippocampus (1.3 × 1.3 × 1.9 mm^3^) (Fig. 1) according to the mouse brain atlas. The static field homogeneity over the voxel was manually adjusted using first- and second-order shimming. Water-suppressed ^1^H MRS was performed using a point-resolved spectroscopy (PRESS) sequence (TR = 3000 ms, TE = 15 ms, NA = 384, number of data points = 2048, spectral bandwidth = 5000 Hz, and scan time = 32 min) [25]. An outer volume suppression module was used before the main sequence, which was interleaved with a water suppression module using variable-power RF pulses with optimized relaxation delays (VAPOR) [26, 27]. A non-water-suppressed spectrum was also acquired for each voxel (NA = 8).

Metabolite quantification was performed using the LC-Model (version 6.3-1L) with the vendor-provided spectral basis set [28]. The absolute concentrations of the following 17 metabolites were estimated: alanine (Ala), aspartate (Asp), creatine (Cr), GABA, glucose (Glc), glutamine (Gln), glutamate (Glu), glutathione (GSH), glycerophosphorylcholine (GPC), lactate (Lac), myo-inositol (Ins), N-acetylaspartate (NAA), N-acetylaspartylglutamate (NAAG), PCr (Phosphocreatine), phosphorylcholine (PCh), scyllo-inositol (Scyllo), and taurine (Tau). Only those results with Cramér-Rao-lower-bounds (CRLB, %SD) ≤ 50% were included in the statistical analysis [29, 30].

### Statistical analysis

Mouse social behaviors tested in 3-chamber test were compared using two-way ANOVA with Sidak’s multiple comparisons test, while the preference indices were analyzed using a two-tailed unpaired t-test with Welch’s correction. Metabolite levels and ratios were also examined using a two-tailed unpaired t-test with Welch’s correction. The correlation coefficient (*r*) between mice social behaviors and levels or ratios of metabolites was calculated with linear regression using the Pearson correlation method. To compute the significance of correlation, we generated empirical null distributions of correlation coefficients by randomly permuting the samples 5000 times. Based on empirical null distributions, we computed adjusted p-values for the Pearson correlation (Pr). Metabolites with Pr < 0.05 were selected as key metabolites. Metabolites with Pr < 0.05 and Pr < 0.025 are marked in the figure described as 95% or 97.5% quantiles, respectively. Differential expression of key metabolites between *Cntnap2*^+/+^ and *Cntnap2*^−/−^ mice were tested using Student’s t-test using customized MATLAB code. For the key metabolites, simple logistic regression classification was carried out to determine the concentration with the highest discriminant power between the two groups. Given the small sample size, all the samples were used to train the model. Specificity, sensitivity, and optimal concentration criteria for discriminating the two groups were assessed using receiver operating characteristic (ROC) curves in the binormal approach, represented by corresponding area under the curve (AUC) values with 95% confidence level. All statistical analyses except the permutation test were performed using the GraphPad Prism 7.00. Mouse social behaviors and preference index are shown as mean ± standard error of the mean (S.E.M.), while the amounts of metabolites or the ratio of different metabolites are shown as mean ± standard deviation (S.D.).

## Results

### *Cntnap2*^−/−^ mice show impaired social behaviors

Mutations in the *Cntnap2* gene are strongly associated with ASD [31]. *Cntnap2*^−/−^ mice show core ASD symptoms, such as reduced vocalization/social interaction and increased repetitive behaviors [32]. To confirm the previously reported social behavior deficits in *Cntnap2*^−/−^ mice, we first tested *Cntnap2*^−/−^ mice in the 3-chamber social behavior test (Fig. 1a). In the social preference test, *Cntnap2*^−/−^ mice showed a comparable level of investigation time towards target conspecifics compared to that for an empty chamber, while *Cntnap2*^+/+^ mice showed a significant preference for target conspecifics (Fig. 1c). Meanwhile, *Cntnap2*^−/−^ mice showed a significantly lower preference index for novel mice compared to *Cntnap2*^+/+^ mice (Fig. 1d). In addition, *Cntnap2*^−/−^ mice did not exhibit a significant preference for novel conspecifics during the social recognition test, whereas *Cntnap2*^+/+^ mice showed a significant preference for novel conspecifics compared to that for familiar conspecifics (Fig. 1e). However, the preference index for novel mice shown by *Cntnap2*^−/−^ mice was not significantly lower than that of *Cntnap2*^+/+^ mice (Fig. 1f). These results suggest that *Cntnap2*^−/−^ mice have a severely impaired social preference, and mildly impaired social recognition. We used *Cntnap2*^−/−^ mice with a confirmed social preference deficit for 9.4T ^1^H-MRS recording, 2 weeks after the 3- chamber social behavior test (Fig. 1b). Metabolites from the prefrontal cortex and hippocampus, which are known to govern social behavior, were examined by 9.4T ^1^H-MRS [12, 33, 34] (Fig. 1g and h). In the raw data set, we found that all the metabolites analyzed showed comparable concentrations between *Cntnap2*^−/−^ and *Cntnap2*^+/+^ mice (Fig. 1i and j, Additional file 1: Tables S1 and S2). We analyze the MRS data more closely in the following section.

### Several brain metabolites level or their ratio show high correlation with mice sociability

**Fig. 2:**
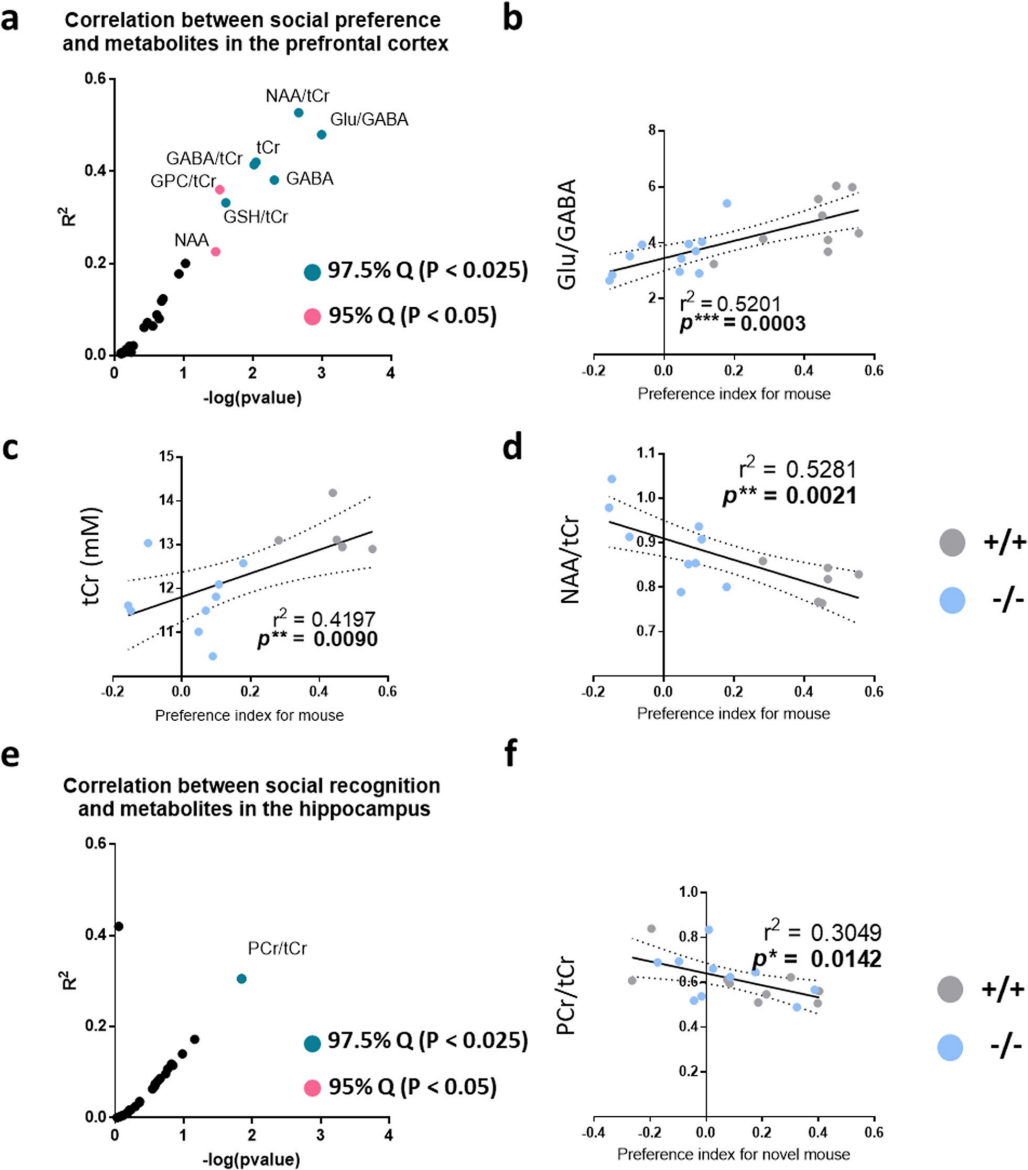
Linear regression analyses between metabolites and rodent social behavior. **a** Volcano plot exhibiting a correlation between social preference and the metabolites in the prefrontal cortex. Metabolites with a significant correlation was colored as pink or cyan depending on the quantile of actual Pearson’s coefficient from the permutated values, 95% (P < 0.05) or 97.5% (P < 0.025) respectively. **b–d** Correlation between mice social preference index and Glu/GABA, tCr or NAA/tCr. Pearson Correlation Coefficients was used to evaluate linear correlations. Each linear regression line is shown with 95% confidence bands (two dotted lines). The degree of significant correlation (*P*) and goodness of fit (r^2^) were written in each figure. **e** Volcano plot exhibiting a correlation between social recognition and metabolites in hippocampus. Metabolites with a significant correlation was colored as pink or cyan depending on the quantile of actual Pearson’s coefficient from the permutated values, 95% or 97.5% respectively. **f** Correlation between mice social preference index for novel mouse and PCr/tCr

Using the metabolites whose concentrations were measured by 9.4T ^1^H-MRS, we analyzed the relationship between metabolite levels and mice sociability. To examine the correlation, mice social preference and social recognition index were compared with each metabolite in the prefrontal cortex and hippocampus (Fig. 2a, see Additional file 1: Table S1 and S2). There were several candidate metabolites in the prefrontal cortex that showed a significant correlation with social preference (Fig. 2a). Notably, there were positive correlations between social preference and the Glu/GABA ratio and tCr level in the prefrontal cortex, even though the levels of metabolite substrates were comparable between groups (Figs. 1i, 2b, and c). In addition, the social preference index was negatively correlated with the prefrontal NAA/tCr ratio (Fig. 2d). However, there was no metabolite in the prefrontal cortex which showed a significant correlation with social recognition (Additional file 1: Fig. S1a). Furthermore, no metabolite in the hippocampus showed a significant correlation with social preference (Additional file 1: Fig. S1b). Consistent with the notion that hippocampal function is crucial for social recognition [33, 34], we found that the PCr/tCr ratio in the hippocampus was negatively correlated with the social recognition ability of mice, but not with the social preference (Fig. 2e, f and Additional file 1: Fig. S2e). Neither the Glu/GABA and NAA/tCr ratios nor tCr levels in the prefrontal cortex showed a significant correlation with social recognition, suggesting that these metabolites in the prefrontal cortex are selectively correlated with social preference, but not social recognition in mice (Additional file 1: Fig. S2a–c).

### *Cntnap2*^−/−^ mice exhibit abnormal metabolic regulation in the prefrontal cortex or in the hippocampus

**Fig. 3:**
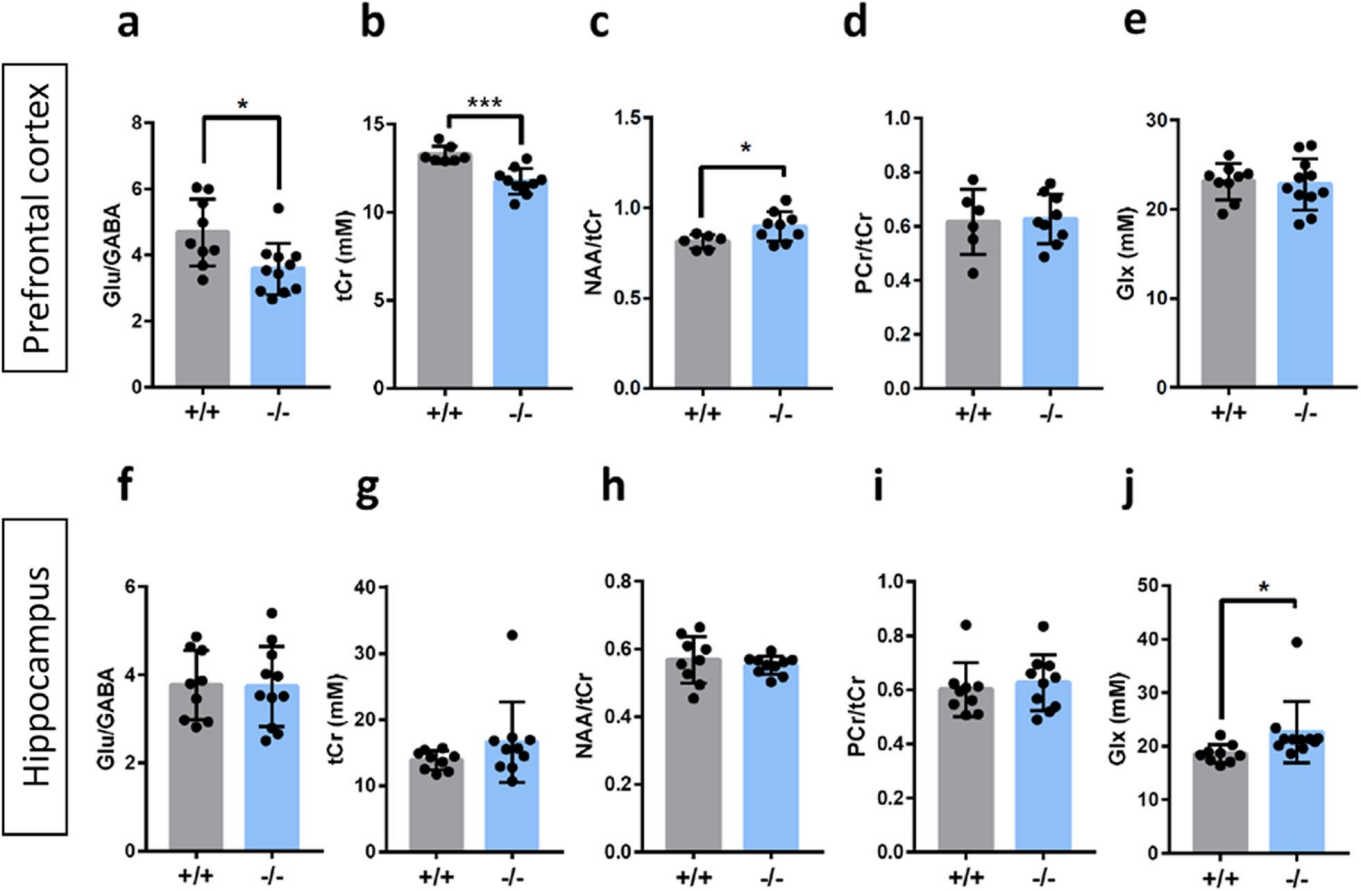
*Cntnap2*^−/−^ mice exhibit altered levels of metabolites in the prefrontal cortex and hippocampus. **a** *Cntnap2*^−/−^ mice showed significantly lower level of Glu/GABA ratio in the prefrontal cortex than *Cntnap2*^+/+^ mice. Two-tailed unpaired t-test with Welch’s correction, *P = 0.0111. **b** *Cntnap2*^−/−^ mice showed significantly lower level of total Creatine (Creatine + phospho − Creatine) in the prefrontal cortex. Two-tailed unpaired t-test with Welch’s correction, ***P = 0.0001. **c** *Cntnap2*^−/−^ mice showed significantly higher level of NAA/tCr ratio in the prefrontal cortex than *Cntnap2*^+/+^ mice. Two-tailed unpaired t-test with Welch’s correction, *P = 0.0220. **d** *Cntnap2*^−/−^ mice showed a comparable level of PCr/tCr ratio in the prefrontal cortex to *Cntnap2*^+/+^ mice. Two-tailed unpaired t-test with Welch’s correction, *P* = 0.8610. **e** *Cntnap2*^−/−^ mice showed a comparable level of Glx (Glutamine + Glutamate) in the prefrontal cortex. Two-tailed unpaired t-test with Welch’s correction, *P* = 0.7958. **f** *Cntnap2*^−/−^ mice showed a comparable level of GABA/Glu ratio in the hippocampus to *Cntnap2*^+/+^ mice. Two-tailed unpaired t-test with Welch’s correction, *P* = 0.8243. **g** *Cntnap2*^−/−^ mice showed a comparable level of total Creatine in the hippocampus to *Cntnap2*^+/+^ mice. Two-tailed unpaired t-test with Welch’s correction, *P* = 0.1994. **h** *Cntnap2*^−/−^ mice showed a comparable level of NAA/tCr in the hippocampus to *Cntnap2*^+/+^ mice. Two-tailed unpaired t-test with Welch’s correction, *P* = 0.5117. **i** *Cntnap2*^−/−^ mice showed a comparable level of PCr/tCr in the hippocampus to *Cntnap2*^+/+^ mice. Two-tailed unpaired t-test with Welch’s correction, *P* = 0.5854. **j** *Cntnap2*^−/−^ mice showed significantly higher level of Glx in the hippocampus to *Cntnap2*^+/+^ mice. Two-tailed unpaired t-test with Welch’s correction, *P = 0.0478

Based on their relationships with social behaviors, metabolites exhibiting significant correlation with sociability, as measured by the social preference index, were compared between genotypes. Interestingly, as its alteration is commonly reported in multiple ASD mouse models including *Cntnap2*^−/−^ mice, the Glu/GABA ratio in the prefrontal cortex was significantly lower in *Cntnap2*^−/−^ mice than *in Cntnap2*^+/+^ mice [14, 32, 35] (Fig. 3a). *Cntnap2*^−/−^ mice also showed significantly lower level of tCr and higher NAA/tCr ratio in the prefrontal cortex compared to *Cntnap2*^−/−^ mice (Fig. 3b and c). In addition, these metabolic changes between genotypes were not observed in the hippocampus (Fig. 3f–h). In addition, although PCr/tCr in the hippocampus showed a significant correlation with the social recognition ability of mice, *Cntnap2*^−/−^ and *Cntnap2*^+/+^ mice showed comparable levels of the PCr/tCr ratio in the hippocampus (Figs. 2f and 3i). The PCr/tCr ratio was also comparable between genotypes in the prefrontal cortex (Fig. 3d). In addition, the levels of Glx, which include glutamate and glutamine, were not significantly different between *Cntnap2*^−/−^ and *Cntnap2*^+/+^ mice in the prefrontal cortex but were significantly higher in the hippocampus of *Cntnap2*^−/−^ mice than *in Cntnap2*^+/+^ mice (Fig. 3e and j). However, there was no correlation between Glx levels and mouse behavior either in the prefrontal cortex or hippocampus (Additional file 1: Fig. S2d and f), suggesting that Glx might not be an ideal biomarker for social behavior.

### Discrimination power of prefrontal Glu/GABA ratio, NAA/tCr ratio or tCr level on *Cntnap2* genotype

**Fig. 4:**
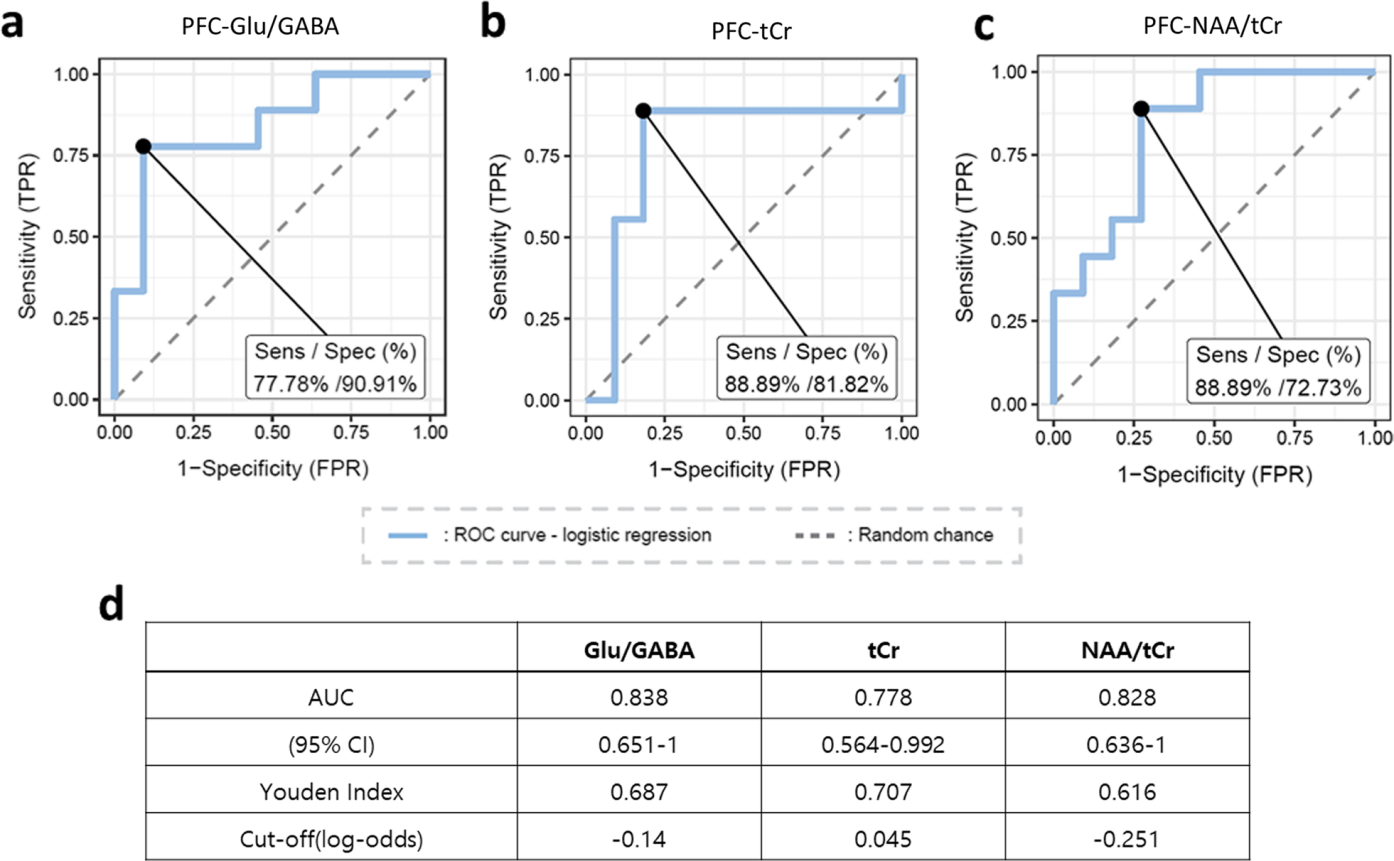
Prefrontal Glu/GABA, tCr, and NAA/tCr precisely predict the genotype. **a–c** ROC curve analyses of metabolites showing a significant correlation with social preference. **d** Information table with detail description for each ROC analysis

To examine the discriminant power of the prefrontal metabolites, we generated a simple logistic regression model and tested it using receiver operating characteristic (ROC) curves. The prefrontal Glu/GABA ratio had an AUC value of 0.838 with a sensitivity of 77.78% and specificity of 90.91%, suggesting that the prefrontal Glu/GABA ratio can be used to properly distinguish *Cntnap2*^+/+^ and *Cntnap2*^−/−^ mice (Fig. 4a and d). Consistent with its comparable level between groups, such a high prediction was not shown using glutamate only (Additional file 1: Fig. S3a and d). Along with the significant difference between groups, tCr level and NAA/tCr ratio also showed significantly accurate predictive scores (Fig. 4b–d). Such metabolic prediction was not precise enough to predict the mouse genotype when using either PCr or NAA alone (Additional file 1: Fig. S3b–d).

## Discussion

Neuroimaging biomarkers such as functional connectivity and brain volume have been introduced as ASD biomarkers [36, 37]. In several human cases with *Cntnap2* mutations, focal malformation of brain regions or reduced long-range connectivity have been reported [38, 39]. Even though MRS has also been used to reveal abnormal levels of metabolites not only in ASD rodent models, but also in humans with ASD [40, 41], patients with *Cntnap2* mutations were scarcely reported for their brain metabolic changes [42]. There were limited studies reported altered metabolites in patients with *Cntnap2* mutant variants [43, 44]. In the present study, we found strong correlations between the social preference index and Glu/GABA, NAA/tCr ratio, and tCr level in the prefrontal cortex of *Cntnap2*^−/−^ mice using in vivo MRS technique that suggests using these metabolites as biomarkers for social preference phenotypes in ASD. Since ASD exhibits high comorbidity with other neurodevelopmental disorders such as intellectual disability, epilepsy, and attention deficit hyperactivity disorder, finding biomarkers for a specific phenotype such as social preference seems to be more reliable, rather than finding biomarkers for individual disorders [45–47].

E/I imbalance has been suggested as one of the mechanisms underlying behavioral phenotypes associated with ASD [14, 15]. Hence, detecting changes in the E/I balance has been suggested as a mechanism-based diagnostic index for ASD [48]. However, it is worth noting that the E/I imbalance has been shown to be brain region-dependent both in humans and mice [35, 49–52]. For example, Horder et al. (2018) reported that Glu concentration was reduced in the striatum, but not in the prefrontal cortex, in humans diagnosed with ASD using MRS [35]. An MRS study using *Nf1*^+/-^ mice, a mouse model of neurofibromatosis type 1, showed increased GABA/Glu ratios in the prefrontal cortex and striatum, but not in the hippocampus [53]. We also found that the Glu/GABA ratio is significantly changed only in the prefrontal cortex in *Cntnap2*^−/−^ mice, highlighting that examining the E/I balance in a region-specific manner is critical for investigating cellular mechanisms or developing diagnostic biomarkers for ASD or other neurodevelopmental disorders. Such a region-specific E/I imbalance in *Cntnap2*^−/−^ mice may be derived not only from the abundancy of *Cntnap2* differ by brain regions, but also from the expression of downstream molecules of *Cntnap2* [32, 54]. Mechanisms underlying brain region-specific E/I imbalance associated with ASD would be an important subject for further investigation.

Even though E/I imbalance has been suggested as a common mechanism for ASD and other neurodevelopmental disorders, the direction of change depends on the conditions. [17, 19]. More interestingly, there are inconsistent results regarding the direction of the E/I ratio changes in *Cntnap2*^−/−^ mice [13, 21]. We found that the E/I ratio measured by the Glu/GABA ratio using MRS was significantly decreased in the prefrontal cortex, but not in the hippocampus of *Cntnap2*^−/−^ mice. This result is consistent with previous ex vivo studies that show a reduction in excitatory synaptic transmission in the prefrontal cortex or in the visual cortex of adult *Cntnap2*^−/−^ mice [20, 55]. In contrast, other studies have suggested reduced inhibition in the cortex of *Cntnap2*^−/−^ mice, suggesting that *Cntnap2* deletion may increase the E/I ratio [13, 32, 56]. These seemingly inconsistent results may stem from subtle differences in experimental conditions, such as the age of the animals. It has been suggested that a discrepancy in E/I ratio can occur at different developmental stages within a single mouse subject [56, 57]. In this study, due to burdensome hours under anesthesia for adolescent mice, we used 20-week-old adult mice for MRS. Longitudinal MRS study in the same mice subjects or human with ASD would provide more information on the dynamic changes of the E/I ratio in multiple brain regions.

It should be noted that the Glu/GABA ratio imbalance assessed by metabolites in this study and the synaptic E/I imbalance reported in previous studies may have different meanings. The decreased Glu/GABA ratio may not be directly translated into the decreased synaptic E/I imbalance since the synaptic E/I balance can be regulated not only by the concentration of neurotransmitters, but also by homeostatic or metaplastic mechanisms such as adjusting the number or activity of postsynaptic receptors [58, 59]. It would be interesting to test whether the manipulation of Glu or GABA actions using their receptor agonists or antagonists can affect mice behavior or cognitive ability in *Cntnap2*^−/−^ mice as shown in other mouse models of neurodevelopmental disorders [60–62].

It is known that ~ 5% of ASD cases are reported for their potential to attribute inborn errors of metabolism [63, 64]. Children with creatine deficiency syndrome show autistic-like features and behaviors, such as developmental delay and intellectual disability [65–67]. Reduction of tCr in children with ASD was also observed not only in the grey matter but also in the white matter with age-dependent variations [68, 69]. Similarly, creatine transporter (Slc6a8) knockout mice exhibited significantly low creatine levels within the brain, resulting in behavioral phenotypes such as hyperactivity and impaired learning and memory, as shown in other ASD mouse models [67]. Together with our finding of decreased total creatine levels in the prefrontal cortex of *Cntnap2*^−/−^ mice, these results suggest that total creatine is also a promising biomarker for social preference deficits in ASD.

Reduced NAA/tCr ratios have been reported in diverse cases, such as patients with Alzheimer’s disease, euthymic bipolar disorder, or men with recreational cannabis usage [70–72]. Interestingly, increased NAA/tCr levels have rarely been reported. Recently, NAA has been commonly discussed as a metabolite that indicates neural dysfunction by various factors, including neuroinflammation [73]. Although further confirmation is essential, based on its unique feature of elevated ratio in this ASD mouse model, high correlation with mouse behavior, and significant alteration by genotype, the NAA/tCr ratio can be suggested as one of the potential biomarkers like the other two metabolic alterations discussed above.

We tested whether the metabolites could be used to predict the *Cntnap2* genotypes by using ROC curve analysis. We found that the prefrontal Glu/GABA ratio, NAA/tCr ratio or tCr level have strong discrimination power on the genotype, suggesting that these metabolites might be useful imaging biomarkers for predicting social deficits associated with ASD. However, using metabolites to diagnose ASD needs to be further confirmed using several other types of biomarkers because dysregulated metabolites could be an epiphenomenon of ASD, which requires additional studies to confirm their correlations [74]. It should also be noted that our results are derived from one mutant mouse line and the relatively small number of animals were used in this study.

## Conclusions

In this study, by using ^1^H-MRS and behavioral tests, we found that the Glu/GABA ratio, NAA/tCr ratio, and tCr level in the prefrontal cortex are significantly correlated with the social preference index of mice. These metabolites were significantly different in *Cntnap2*^−/−^ mice compared to those in wild-type mice. Moreover, these metabolites showed high specificity and selectivity in discriminating *Cntnap2* mutant from wild-type mice. Our study suggests that the accuracy of diagnosing ASD might be substantially improved by using multimodal approaches such as the non-invasive MRS technique.

## Supplementary Information

**Additional file 1.** **Table S1.** Total metabolites in the prefrontal cortex. **Table S2.** Total metabolites in the hippocampus. **Supplementary figure S1.** Volcano plot showing significant Pearson’s correlation between metabolites and rodent sociability. **Supplementary figure S2.** Other linear regression analyses between metabolites and rodent sociability. **Supplementary figure S3.** ROC analyses between metabolites and rodent genotype.

## Data Availability

All data generated or analyzed in this study are included in this published article.

## Abbreviations

Ala: Alanine
ASD: Autism spectrum disorder
Asp: Aspartate
Cr: Creatine
GABA: γ-Aminobutyric acid
Gln: Glutamine
Glu: Glutamate
GPC: Glycerylphosphorylcholine
GSH: Glutathione
Glx: Glutamate + glutamine
HPC: Hippocampus
Ins: Myo-inositol
mEPSC: Miniature excitatory postsynaptic currents
MM: Macromolecule
MRI: Magnetic resonance imaging
MRS: Magnetic resonance spectroscopy
NAA: N-acetylaspartate
NAAG: N-acetylaspartylglutamate
PCr: Phosphocreatine
PCh: Phosphorylcholine
PFC: Prefrontal cortex
ROC: Receiver operating characteristics
scyllo: Scyllo-inositol
tau: Taurine
tCr: Total creatine
tCho: PCh + GPC
tNAA: NAA + NAAG
