# Supplementary material for: Decreased in vivo glutamate/GABA ratio correlates with the social behavior deficit in a mouse model of autism spectrum disorder
Source: Mol Brain. 2022 Feb 19;15:19. doi: 10.1186/s13041-022-00904-z (PMC8858545; doi:10.1186/s13041-022-00904-z)
Supplement: Supplementary file 1 — Additional file 1. Table S1. Total metabolites in the prefrontal cortex. Table S2. Total metabolites in the hippocampus. Supplementary figure S1. Volcano plot showing significant Pearson’s correlation between metabolites and rodent sociability. Supplementary figure S2. Other linear regression analyses between metabolites and rodent sociability. Supplementary figure S3. ROC analyses between metabolites and rodent genotype. [file 13041_2022_904_MOESM1_ESM.pdf]

List of contents

Table S1. Total metabolites in the prefrontal cortex

Table S2. Total metabolites in the hippocampus

Supplementary figure 1. Volcano plot showing significant Pearson's correlation between metabolites and rodent sociability

Supplementary figure 2. Other linear regression analyses between metabolites and rodent sociability

Supplementary figure 3. ROC analyses of the prefrontal metabolites

Table S1. Total metabolites in the prefrontal cortex

PFC	Ala	Ala %SD	Asp	Asp %SD	Cr	Cr %SD	PCr	PCr %SD	GABA	GABA %SD	Glc	Glc %SD	Gln	Gln %SD	Glu	Glu %SD	GPC	GPC %SD	PCh	PCh %SD	GSH	GSH %SD	Ins	Ins %SD	Lac	Lac %SD	NAA	NAA %SD	NAAG	NAAG %SD	Scyllo	Scyllo %SD	Tau	Tau %SD
WT1	1.136	81	5.331	26	1.838	116	12.691	16	2.375	52	2.058	42	6.186	18	14.332	8	2.959	7	0	999	4.963	13	9.979	10	0	999	8.981	9	0.98	66	0	999	23.41	5
WT2	1.874	35	3.838	25	0	999	9.697	6	2.153	41	0.122	499	6.587	14	12.904	7	1.748	28	0.545	98	4.54	10	4.962	12	0	999	7.692	8	0.249	198	0	999	14.549	5
WT3	1.745	38	7.247	16	5.694	18	8.492	13	3.295	28	0.515	153	5.449	16	18.347	5	0.606	91	2.427	24	4.41	11	11.537	7	0	999	10.876	6	0.79	69	0	999	23.742	4
WT4	2.411	22	7.973	13	2.988	39	10.129	13	3.599	21	0.701	100	8.153	9	17.92	4	1.429	31	1.169	40	4.844	8	8.519	6	1.531	36	10.02	5	1.309	33	0	999	19.073	3
WT5	2.432	19	7.167	14	5.81	14	7.158	11	4.736	16	1.09	64	6.507	12	17.464	4	1.089	38	1.749	25	4.36	9	11.007	5	0	999	10.612	5	1.148	35	0	999	21.899	3
WT6	2.164	25	8.722	13	4.027	36	8.915	18	4.403	19	0	999	9.114	9	18.069	4	1.669	27	1.584	30	5.213	8	9.317	6	0	999	10.917	5	0.494	112	0	999	19.245	3
WT7	1.768	38	8.553	12	7.413	11	5.492	16	4.195	20	0.83	88	4.776	16	18.238	4	1.332	34	1.805	27	4.626	9	10.248	6	0	999	10.695	5	1.347	35	0	999	21.497	4
WT8	2.188	27	8.224	13	0.349	394	13.376	12	5.225	16	0	999	5.986	13	16.963	4	1.43	32	1.326	36	5.166	8	11.942	5	0	999	11.428	5	0.768	63	0	999	26.235	3
WT9	1.923	35	7.107	16	4.472	28	8.632	15	4.509	20	0.28	275	4.985	18	18.684	5	1.183	43	1.739	31	4.412	11	10.763	6	2.182	29	11.252	6	0.333	171	0	999	23.975	3
KO1	0.432	187	5.722	18	5.648	20	5.368	21	3.927	23	0.161	413	10.289	9	13.494	6	1.601	31	1.069	49	5.018	10	6.82	9	0	999	8.692	6	0.13	378	0	999	16.977	4
KO2	1.683	42	6.212	16	3.091	39	7.366	17	3.668	23	0.542	125	5.329	16	13.608	6	1.277	38	1.045	50	4.64	10	8.952	7	0	999	8.933	6	0	999	0	999	20.382	4
KO3	2.186	30	7.396	15	5.665	22	6.43	19	4.282	21	0.78	94	4.313	20	17.281	5	1.583	30	1.343	38	4.398	10	9.195	7	0	999	10.975	6	0.0753	740	0	999	21.384	4
KO4	3.237	15	6.269	16	3.043	33	9.537	11	2.513	32	0.282	247	4.729	16	13.605	6	0.606	77	1.724	28	4.521	9	10.58	6	0	999	10.073	5	0	999	0	999	21.997	3
KO5	2.055	28	8.028	13	5.152	23	7.888	15	5.135	15	0.872	84	5.417	14	18.129	4	1.697	25	1.8	25	4.816	8	9.747	6	0	999	11.909	4	1.003	46	0	999	22.126	3
KO6	2.805	24	9.145	14	1.412	129	13.512	15	5.244	19	1.368	63	3.883	21	20.621	4	1.038	58	2.305	27	5.438	9	12.109	6	0	999	12.227	5	1.171	48	0	999	26.66	3
KO7	1.75	30	6.368	16	5.102	17	6.712	13	5.718	13	1.146	61	4.791	16	16.638	5	2.061	20	0.83	52	4.823	8	9.726	6	0	999	11.061	5	0.945	43	0	999	20.591	4
KO8	1.336	37	7.516	13	3.124	30	8.375	13	4.126	18	0.163	396	5.555	13	16.352	4	1.456	29	1.184	37	4.661	9	8.44	7	0	999	9.797	5	0.353	115	0	999	21.382	3
KO9	2.774	23	8.032	13	1.71	89	10.152	15	5.603	15	0	999	5.695	14	16.67	5	1.46	39	1.565	39	4.448	10	8.748	7	8.5	8	12.778	4	0	999	0	999	21.753	4
KO10	2.021	36	6.735	17	4.471	24	7.145	15	5.825	16	0.196	381	9.499	9	15.5	6	1.538	31	1.275	40	5.677	9	6.518	10	0	999	11.37	5	0	999	0	999	19.8	5
KO11	1.941	32	8.684	12	4.121	26	7.378	16	6.293	12	0.3	223	8.987	9	17.984	4	1.608	27	1.117	40	4.386	9	7.159	7	3.238	19	11.997	4	0.7	61	0	999	17.807	4

Table S2. Total metabolites in the hippocampus

HPC	Ala	Ala %SD	Asp	Asp %SD	Cr	Cr %SD	PCr	PCr %SD	GABA	GABA %SD	Glc	Glc %SD	Gln	Gln %SD	Glu	Glu %SD	GPC	GPC %SD	PCh	PCh %SD	GSH	GSH %SD	Ins	Ins %SD	Lac	Lac %SD	NAA	NAA %SD	NAAG	NAAG %SD	Scyllo	Scyllo %SD	Tau	Tau %SD
WT1	1.788	37	5.639	17	5.809	13	9.614	8	4.137	18	0.886	71	4.48	17	15.741	5	0.564	59	0.952	36	3.355	12	7.881	7	0	999	8.746	5	2.085	18	0	999	18.904	3
WT2	1.559	32	4.711	17	5.33	14	6.813	11	3.48	18	1.416	37	6.701	10	12.031	5	0.417	74	1.11	28	4.032	9	5.04	9	0	999	5.513	7	1.74	21	0	999	13.773	4
WT3	2.207	24	3.218	27	5.309	15	6.418	14	4.121	18	1.137	48	4.084	18	12.257	6	1.536	8	0	999	2.845	13	6.848	7	0	999	7.138	6	1.576	24	0	999	16.384	3
WT4	1.548	34	4.306	19	4.897	16	7.595	11	2.4	28	1.618	34	6.689	10	11.686	5	0.588	55	0.899	37	4.442	8	4.826	9	0	999	6.568	7	1.849	21	0	999	13.583	4
WT5	1.479	32	5.308	17	7.117	13	7.326	13	4.43	16	0.952	67	4.899	14	12.462	5	0.441	85	1.366	28	4.232	9	8.567	6	0.1	614	7.139	6	1.576	24	0	999	18.802	3
WT6	1.559	33	6.614	14	7.026	12	7.321	11	5.465	12	0.434	140	8.362	8	13.699	5	0.459	74	1.508	23	5.057	8	6.18	7	0.982	56	8.595	5	1.842	20	0	999	17.853	3
WT7	2.345	22	7.376	13	6.031	16	8.857	12	3.158	22	0.854	75	4.567	15	14.391	5	0.803	47	1.29	30	4.071	9	8.795	6	2.266	20	8.267	5	1.498	23	0	999	18.662	3
WT8	3.393	16	5.073	18	5.283	19	8.328	13	3.31	22	0.88	67	4.263	17	12.803	5	0.637	52	0.977	35	3.517	11	7.561	7	0	999	8.78	6	0.757	55	0	999	18.596	3
WT9	2.007	25	8.355	12	2.51	45	13.182	10	4.577	15	0.77	86	4.824	13	13.43	5	0.145	274	1.576	25	4.514	8	8.329	6	0	999	10.416	5	0.843	48	0	999	21.637	3
KO1	2.051	23	7.182	12	4.846	17	8.052	12	5.191	13	0.532	109	9.612	7	13.798	4	0.52	57	1.506	19	4.364	8	5.021	9	2.781	18	7.663	5	2.018	14	0	999	15.522	3
KO2	2.82	18	8.57	12	2.792	48	14.139	11	3.847	20	0.763	81	5.391	14	15.468	4	0.734	53	1.27	31	4.871	8	8.346	7	1.306	44	9.018	6	1.546	29	0	999	21.717	3
KO3	1.419	38	7.344	14	6.128	14	11.192	9	3.096	26	0	999	4.524	17	16.726	4	0.703	55	1.541	25	4.395	9	8.747	6	0.625	104	8.697	5	0.825	46	0	999	20.577	3
KO4	3.366	32	14.387	14	10.21	23	22.575	12	6.893	22	2.015	66	8.744	17	30.702	4	0.761	85	3.085	23	8.315	9	16.139	6	2.806	43	18.549	5	2.219	38	0	999	39.354	3
KO5	1.83	24	6.509	14	6.777	11	8.872	9	3.697	19	0.272	245	6.769	10	14.67	5	0.557	67	1.569	25	4.276	9	8.459	6	0	999	8.607	5	1.149	31	0	999	21.338	3
KO6	1.767	27	6.351	14	6.96	9	7.514	8	3.114	22	2.244	28	5.668	12	14.44	5	0.532	63	1.316	26	3.075	11	7.785	6	1.862	26	8.256	5	1.486	21	0	999	18.686	3
KO7	1.044	55	5.741	16	3.906	26	8.854	13	4.678	16	1.598	36	6.501	11	13.071	5	1.955	6	0	999	3.978	10	6.852	7	0.893	58	6.615	7	1.856	22	0	999	17.914	4
KO8	1.89	29	6.058	17	7.916	11	7.577	13	4.357	17	0	999	5.981	13	15.323	5	0.947	42	1.219	34	4.212	9	9.108	6	0	999	8.545	6	2.26	17	0	999	19.961	3
KO9	1.641	33	7.257	14	5.701	18	11.113	10	4.421	18	0.55	128	5.702	13	15.609	5	0.686	63	1.465	30	4.433	9	8.532	6	2.165	27	9.548	5	0.578	74	0	999	22.627	3
KO10	2.01	26	4.248	21	2.31	57	10.361	14	2.495	29	0.585	96	9.258	7	11.972	5	0.314	133	1.272	33	4.374	9	3.921	12	0	999	7.92	6	0.171	290	0	999	13.97	4
KO11	1.201	33	4.154	17	4.952	11	5.762	9	3.412	17	0.627	77	6.737	9	11.903	5	0.962	30	0.405	73	3.44	9	4.687	8	0	999	6.006	6	1.606	17	0	999	12.478	4

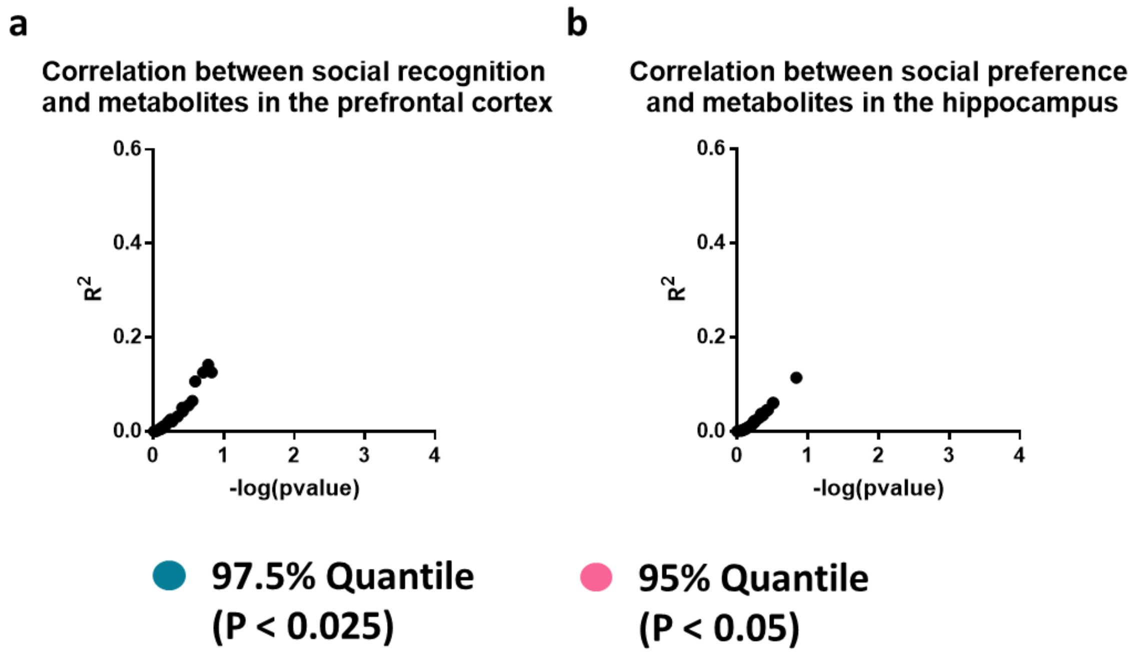

Supplementary figure 1. Volcano plot showing significant Pearson's correlation between metabolites and rodent sociability

Correlation between mice social preference index for novel mouse or social preference index for mouse and metabolites in the prefrontal cortex **(a)** and hippocampus **(b)** was calculated, respectively. Metabolites with a significant correlation was colored as pink or cyan depending on the quantile of actual Pearson's coefficient from the permutated values, 95% or 97.5% respectively.

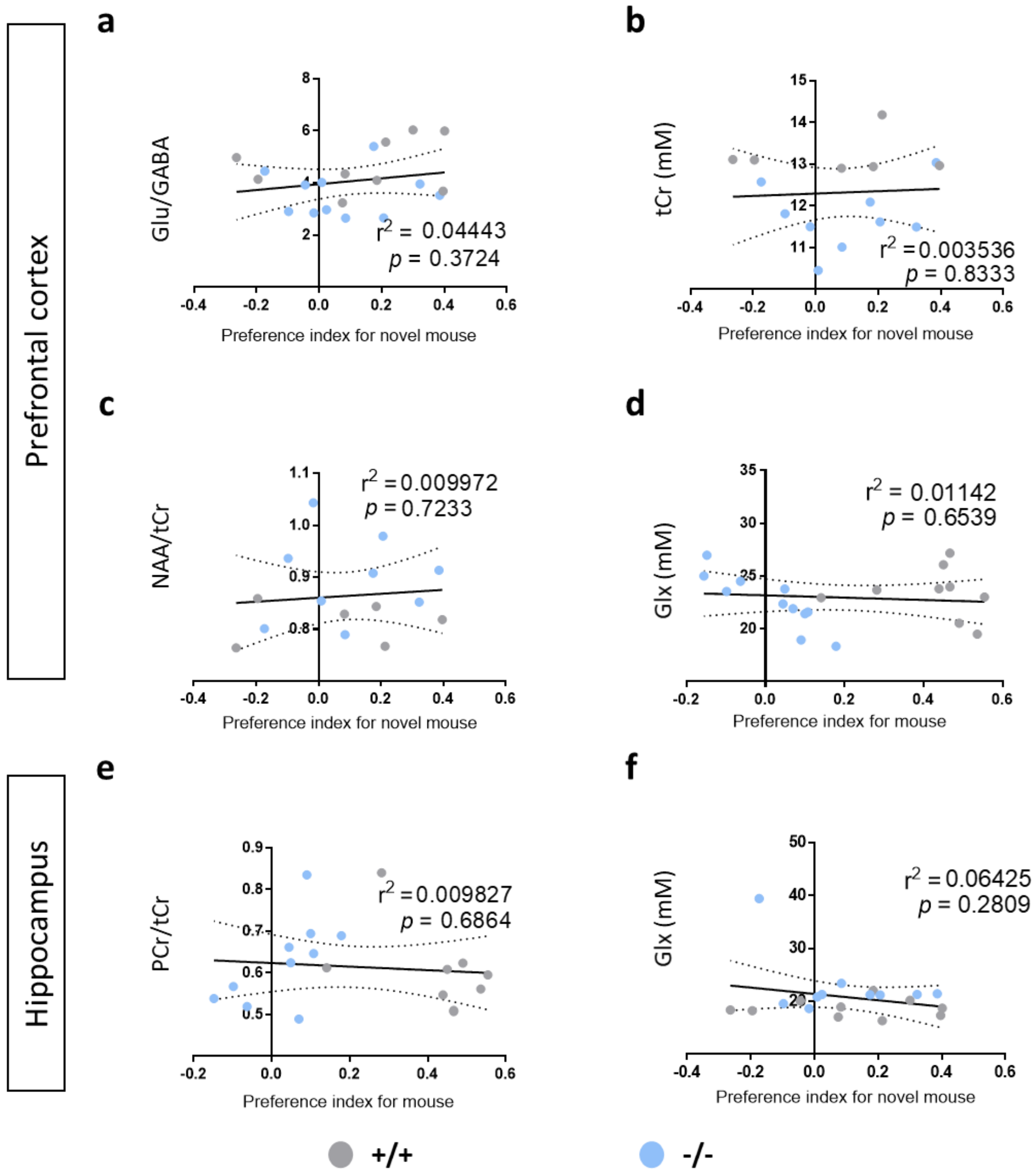

Supplementary figure 2. Other linear regression analyses between metabolites and rodent sociability

(a-c) Correlation between mice social preference index for novel mouse and each of the significantly altered metabolites in the prefrontal cortex was calculated.

(d) Correlation between mice social preference index and Glx, a metabolite significantly altered in the hippocampus was calculated.

(e) Correlation between mice social preference index and PCr/tCr ratio in hippocampus was calculated.

(f) Correlation between mice social preference index for novel mouse and Glx level in hippocampus was calculated.

Each linear regression line is shown with 95% confidence bands (two dotted lines). The degree of significant correlation (*p*) and goodness of fit (r^2) were written in each figure.

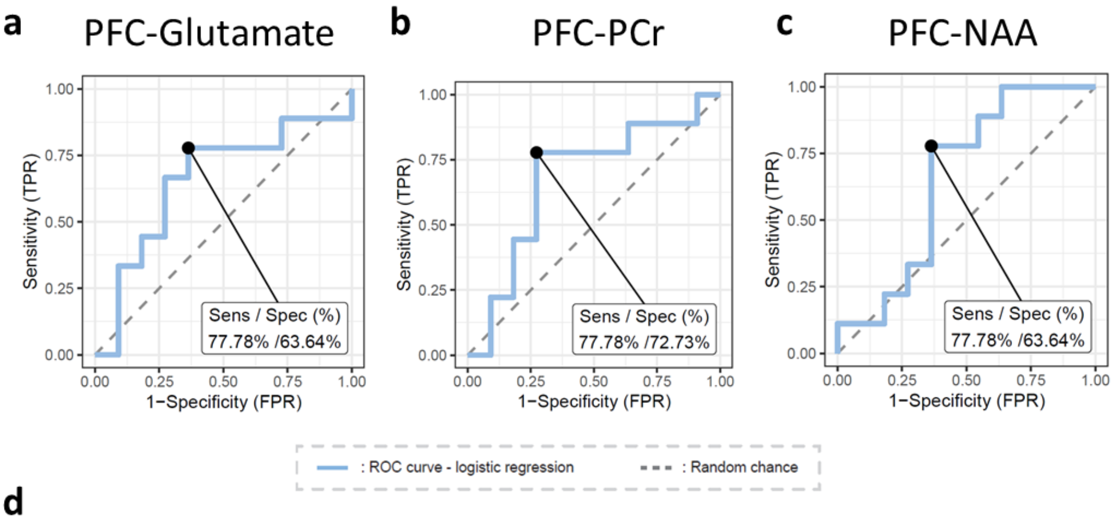

d

	Glu	PCr	NAA
AUC	0.657	0.677	0.657
(95% CI)	0.409-0.904	0.433-0.92	0.409-0.904
Youden Index	0.414	0.505	0.414
Cut-off(log-odds)	-0.156	-0.262	-0.329

Supplementary figure 3. ROC curve analyses of the prefrontal metabolites

(a-c) ROC curve analyses of glutamate (Glu), phosphocreatine (pCr), and N-acetylaspartate (NAA) in the prefrontal cortex. Each metabolic substrate is not precise enough to predict the genotype. **(d)** A table describe a detail information for each ROC analysis.
